# *Spata7* is required for maintenance of the retinal connecting cilium

**DOI:** 10.1038/s41598-022-09530-0

**Published:** 2022-04-02

**Authors:** Jiaxiong Lu, Kaitlyn Xiong, Xinye Qian, Jongsu Choi, Yoon-Kyung Shim, Jacob Burnett, Graeme Mardon, Rui Chen

**Affiliations:** 1 Department of Biochemistry and Molecular Biology, Baylor College of Medicine, Houston, TX USA; 2 Department of Molecular and Human Genetics, Human Genome Sequencing Center, Baylor College of Medicine, Houston, TX USA; 3 Department of Pathology, Baylor College of Medicine, Houston, TX USA

**Keywords:** Neurodegeneration, Cell death, Stress signalling

## Abstract

*SPATA7*, an early onset LCA3 retinal disease gene, encodes a putative scaffold protein that is essential for the proper assembly of the connecting cilium (CC) complex in photoreceptors. Previous studies have shown that SPATA7 interacts with other photoreceptor-specific ciliary proteins, such as RPGR and RPGRIP1, and maintains the integrity of CC integrity. However, although it is known that *Spata7* is required for early formation of the CC, it is unclear if Spata7 is also required for the maintenance of the CC. To investigate *Spata7* function in the retina at the adult stage, loss of function was induced in the adult retina upon tamoxifen induction of an inducible *Spata7* knockout allele (*Spata7*^*flox/−*^; *UbcCreERT2/*^+^). The phenotype of mutant retina was characterized by a combination of histology, immunobiochemistry, and electroretinography (ERG). Our results demonstrated that *Spata7* is also essential for maintaining the integrity of the mature retinal CC. Loss of *Spata7* in adults caused phenotypes similar to those seen in germline mutant mice, including photoreceptor cell degeneration and defective ERG responses. Close examination of the CC revealed significantly shortened NPHP1 length as a result of *Spata7* deletion. Furthermore, mislocalization of rhodopsin, leading to ER stress-mediated apoptosis, was observed in the retinal layers. Our results indicate that *Spata7* is required not only for the establishment but also for the maintenance of the CC of photoreceptors.

## Introduction

Retinal photoreceptor cells contain a primary cilium known as the connecting cilium (CC)^1^. The CC is a slender structure that connects the outer (OS) and inner segments (IS) of photoreceptors and mediates a critical protein trafficking function^2,3^. Mutations in several photoreceptor-specific and common cilia genes can lead to deficient morphogenesis and/or dysfunction of the CC and result in retinal ciliopathies, a group of inherited retinal degenerative diseases including Retinitis pigmentosa (RP)^4^ and Leber congenital amaurosis (LCA)^1^.

Proper formation and maintenance of the CC is essential for proper function of photoreceptor cells. Many genes that are important for CC development are also essential for CC maintenance. For example, both early and late loss of function of intraflagellar transport factors KIF3A or IFT88 result in CC defects and photoreceptor degeneration^5^. In contrast, it has been observed that although NPHP1 is crucial during OS development, once the CC is established, a low level of NPHP1 is sufficient to maintain the proper function of the CC^6^.

We previously identified *SPATA7* (spermatogenesis-associated protein 7) as a disease gene in the *LCA3* locus^7,8^. Mechanistic studies indicate that Spata7 is critical for the integrity of microtubule filaments of the CC and physically interacts with multiple members of the RPGR and NPHP complexes^9^. Loss-of-function mutations in *SPATA7* cause rapid photoreceptor degeneration and lead to LCA in humans^8,10,11^. Similarly, severe early-onset photoreceptor degeneration is observed in *Spata7* germline knockout (*Spata7*^*−/−*^) mouse retinas with 50% of photoreceptors actively undergoing apoptosis by one month of age^12,13^. Loss of SPATA7 protein disrupts the protein trafficking process, leading to ectopic accumulation of proteins within the inner segment of photoreceptor cells and subsequently triggering photoreceptor apoptosis^9,12^.

However, it is unclear if SPATA7 is continuously required for CC maintenance or if its requirement diminishes in mature photoreceptors similar to NPHP1. Using a tamoxifen inducible *Spata7* conditional knockout mouse model (tamoxifen injected *Spata7*^*flox/−*^; *UbcCreERT2*/^+^, or *Spata7 iKO*) to delete *Spata7* in adult mouse retinas, we show that *Spata7* is continuously required for the integrity of the CC of photoreceptor cells. Conditional knockout of *Spata7* in the adult retina causes rapid retinal degeneration defects. In addition, consistent with observations in germline *Spata7*^*−/−*^ mice, photoreceptor cells of tamoxifen-induced *Spata7 iKO* mice exhibit rhodopsin mislocalization in the IS and outer nucleus layer (ONL) and ER stress activation. Taken together, our results indicate that *Spata7* is required for both the initial establishment and continued maintenance of the retinal CC.

## Results

### *UbcCreERT2* successfully deletes* Spata7* in adult mouse retinas

**Figure 1:**
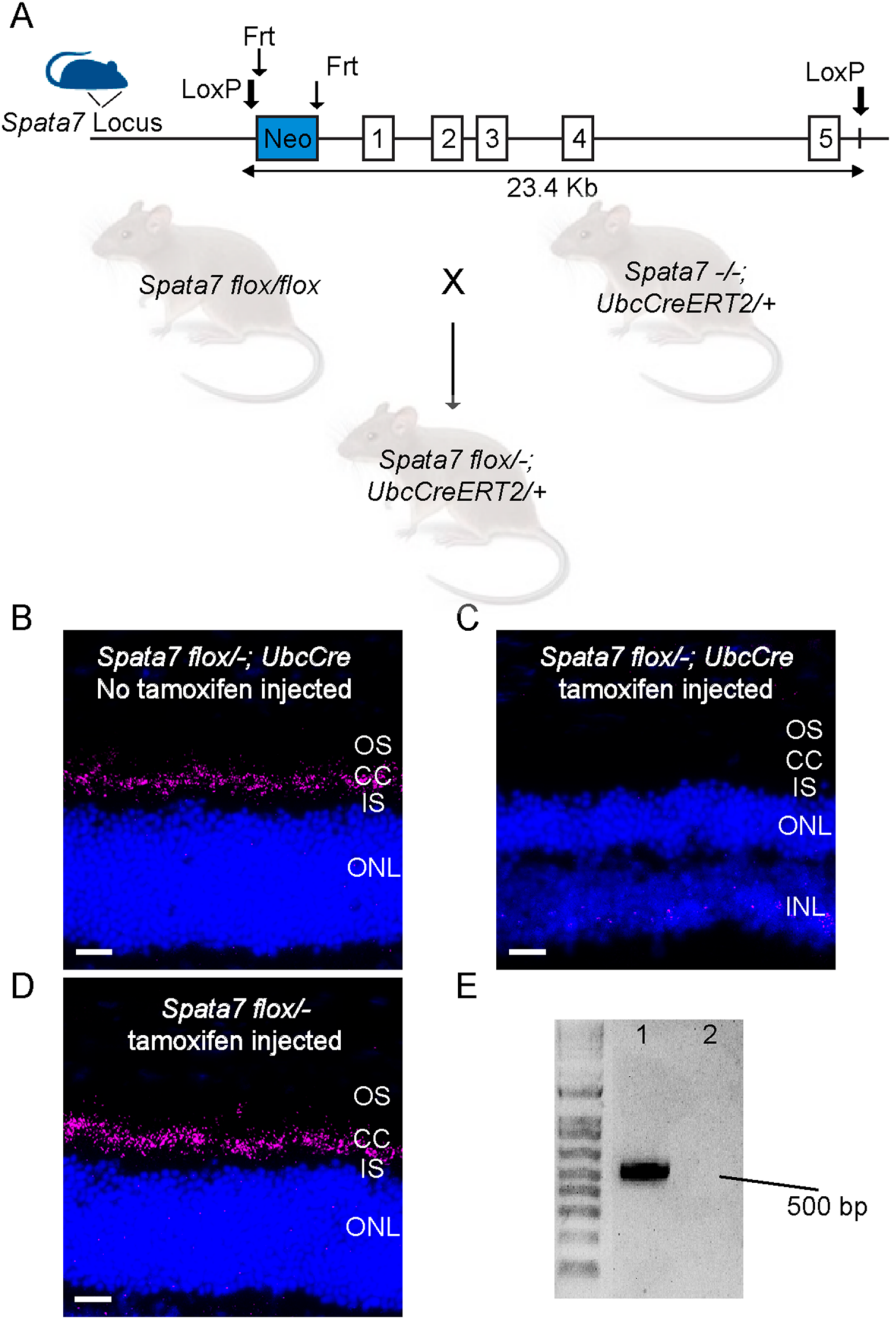
Loss of SPATA7 expression in retinas of *Spata7*^*flox/−*^; *UbcCreERT2/*^+^ mice. (**A**) Schematic of the *Spata7 flox* allele and breeding scheme for generating *Spata7*^*flox/−*^; *UbcCreERT2* mice. Numbered rectangles represent *Spata7* exons. (**B**–**D**) Anti-SPATA7 staining (pink) of retinal cryosections showing absence of Spata7 protein in 4-month-old *Spata7*^*flox/−*^; *UbcCreERT2/*^+^ mice with tamoxifen (injected at 3 months of age) (**C**) compared to non-injected *Spata7*^*flox/−*^; *UbcCreERT2/*^+^ mice (**B**) and tamoxifen-injected *Spata7 *^*flox/−*^ mice (**D**). Nuclei are stained with DAPI (blue). *INL* inner nuclear layer, *IS* inner segment, *ONL* outer nuclear layer, *OS* outer segment, *CC* connecting cilium. (**E**) PCR amplification of genomic DNA shows complete deletion of the floxed portion of *Spata7* following tamoxifen induction in *Spata7*^*flox/−*^; *UbcCreERT2* mice. (1) Non-injected *Spata7*^*flox/−*^; *UbcCreERT2* and (2) tamoxifen injected *Spata7*^*flox/−*^; *UbcCreERT2*. Scale bar = 20 µm.

Previous studies have focused on investigating the role of SPATA7 in the developing retina using germline *Spata7* knockout mice^9,12^. To determine the role of SPATA7 in maintaining the photoreceptor CC in the mature mammalian retina, we generated an inducible *Spata7* knockout mouse (*Spata7*^*flox/−*^; *UbcCreERT2*/^+^) by cross-breeding conditional *Spata7* knockout mice (*Spata7*^*flox/flox*^)^14^ with germline *Spata7* knockout mice harboring the *UbcCreERT2* transgene^15,16^ (*Spata7*^*−/−*^; *UbcCreERT2*/^+^) (Fig. 1A). Cre expression was induced by injecting tamoxifen at 3 months of age, when mouse retinas have fully matured^17^. Previous studies have shown that Spata7 localizes to the CC, which connects the IS and the OS^12^. *UbcCreERT2*-mediated *Spata7* deletion was evaluated by staining for Spata7 protein in the CC of *Spata7*^*flox/−*^; *UbcCreERT2*/^+^ mice one month after tamoxifen induction. Immunostaining showed the absence of Spata7 in the CC of tamoxifen-injected *Spata7*^*flox/−*^; *UbcCreERT2*/^+^ mice, whereas Spata7 protein was readily detected in control mice, including *Spata7*^*flox/−*^; *UbcCreERT2*/^+^ mice without tamoxifen induction and *Spata7*^*flox/−*^ mice without the *UbcCreERT2* allele (Fig. 1B–D). We performed PCR on genomic DNA extracted from isolated mouse retinas using primers flanking the *Spata7* allele^14^, and no band corresponding to was detected for tamoxifen-injected *Spata7*^*flox/−*^; *UbcCreERT2*/^+^ mice compared with control mice (Fig. 1E). These data indicate that the *Spata7* conditional allele was completely deleted in adult mouse retinal cells upon tamoxifen induction, making the *Spata7*^*flox/−*^; *UbcCreERT2*/^+^ mouse a suitable model for studying Spata7 function in adult retina.

### Conditional knockout of *Spata7* in adult mouse retina leads to photoreceptor degeneration

Germline loss of *SPATA7* leads to photoreceptor degeneration in both humans and mice^8,9,12^. To determine the effects of *Spata7* deletion in adult mice, retinas of tamoxifen-induced *Spata7*^*flox/−*^; *UbcCreERT2*/^+^ mice were characterized by functional and morphological studies.

**Figure 2:**
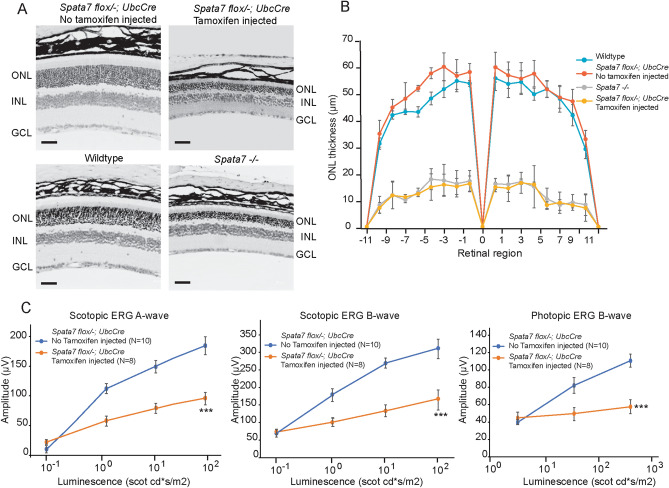
Significant photoreceptor degeneration due to loss of *Spata7* in adult mouse retina. (**A**) Hematoxylin and eosin staining of paraffin-embedded retinal sections shows reduced ONL thickness in 6-month-old *Spata7*^*flox/−*^; *UbcCreERT2* mice following tamoxifen induction at 3 months of age. Tamoxifen non-injected *Spata7*^*flox/−*^; *UbcCreERT2* mice and 3-month-old wild-type and *Spata7*^*−/−*^ mice act as controls. *ONL* outer nuclear layer, *INL* inner nuclear layer, *GCL* ganglion cell layer. (**B**) Spider plot showing retinal morphometry reveals reduced ONL thickness resulting from loss of *Spata7* either in the germline or in adults. The thickness of the ONL was measured at 20 equally spaced positions along the vertical meridian of the retina. Position 0 corresponds to the optic nerve head. Each point represents the mean ± SEM obtained for each group (n ≥ 3 mouse retinas). (**C**) 6-month-old *Spata7*^*flox/−*^; *UbcCreERT2* mice with tamoxifen induction at 3 months of age exhibit reduced ERG scotopic a-wave and b-wave and photopic b-wave amplitudes compared to non-injected controls. Statistical analysis was performed using the Mann–Whitney test (***p < 0.001). N = 10 and N = 8 for *Spata7*^*flox/−*^; *UbcCreERT2* mice without or with tamoxifen induction, respectively.

Hematoxylin and eosin (H&E) staining was performed on retinal sections of *Spata7*^*flox/−*^; *UbcCreERT2*/^+^ mice three months after induction. H&E results indicated that the induced conditional knockout mice showed approximately 70% reduction in ONL thickness compared to control mice (Fig. 2A). Photoreceptor degeneration across the entire retina was measured by retinal morphometry. Similar to *Spata7*^*−/−*^ mice, tamoxifen-injected *Spata7*^*flox/−*^; *UbcCreERT2*/^+^ mice also presented with severely reduced ONL thickness (70% in tamoxifen-injected *Spata7*^*flox/−*^; *UbcCreERT2*/^+^ mice and 65% in *Spata7*^*−/−*^ mice) compared to wildtype and non-injected *Spata7*^*flox/−*^; *UbcCreERT2*/^+^ mice (Fig. 2B).

Consistent with the morphological changes described above, electroretinograms (ERGs) performed on tamoxifen-injected *Spata7*^*flox/−*^; *UbcCreERT2*/^+^ mice 3 months after induction showed a significant reduction in the amplitudes of both scotopic and photopic waves in tamoxifen-induced *Spata7*^*flox/−*^; *UbcCreERT2*/^+^ mice compared to controls (Fig. 2C). These data indicate that the loss of both cone and rod photoreceptors in the induced adult *Spata7* knockout mice causes impaired photoresponsiveness. Collectively, these results demonstrate that, similar to what has been observed in germline mutant retinas, loss of *Spata7* in adult mouse retinas leads to photoreceptor cell degeneration.

### Protein mis-localization and ER-tress activation in *Spata7* adult knockout mouse retina

**Figure 3:**
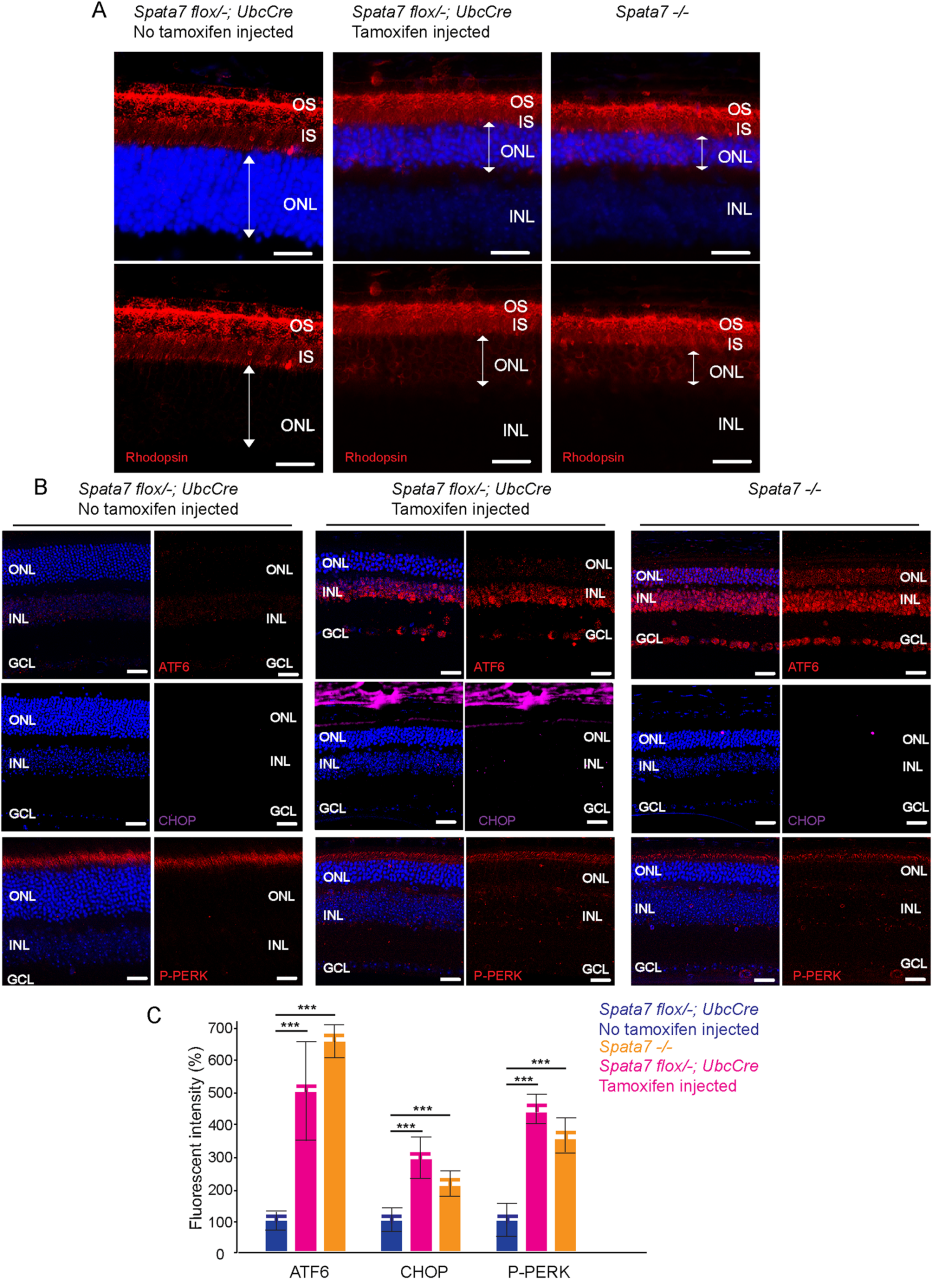
Rhodopsin mislocalization and ER stress activation in *Spata7*^*flox/−*^; *UbcCreERT2* mice. (**A**) Mislocalized rhodopsin is observed in retinal sections of tamoxifen injected *Spata7*^*flox/−*^; *UbcCreERT2* and *Spata7*^*−/−*^ mice compared with tamoxifen non-injected *Spata7*^*flox/−*^; *UbcCreERT2* retinas. Scale bar = 5 µm. (**B**) Elevated ATF6, CHOP, and P-PERK signals were detected in the retina of tamoxifen injected *Spata7*^*flox/−*^; *UbcCreERT2* mice, while little to no signal was detected in tamoxifen non-injected *Spata7*^*flox/−*^; *UbcCreERT2* mouse retina. Scale bar = 20 µm. (**C**) Quantification of the fluorescence intensities of ATF6, CHOP, and P-PERK from the whole retina indicates increased ER stress activation in tamoxifen injected *Spata7*^*flox/−*^; *UbcCreERT2* mice and *Spata7*^*−/−*^ mice. Fluorescence intensity is reported relative to the signal of each marker in non-injected *Spata7*^*flox/−*^; *UbcCreERT2* retinas. Error bars represent SEM (N = 5 mice per genotype). *OS* outer segment, *IS* inner segment, *ONL* outer nuclear layer, *INL* inner nuclear layer, *GCL* ganglion cell layer.

Previous studies have indicated that the integrity of the CC is lost in germline *Spata7* mutants, resulting in defective protein trafficking, ectopic accumulation of protein in the IS of photoreceptor cells, and ER stress-mediated apoptosis^12^. To test if a similar phenotype is observed in adult *Spata7* knockout mice, immunofluorescence staining was performed to assess the levels of rhodopsin mis-localization and ER stress activation. Substantial mislocalization of rhodopsin to the IS and ONL was observed in the induced *Spata7*^*flox/−*^; *UbcCreERT2*/^+^ mice compared to the control mice (Fig. 3A). Moreover, elevated expression of C/EBP homologous protein (CHOP)^18,19^, activating transcription factor 6 (ATF6)^20,21^, and phospho-pancreatic ER kinase (P-PERK)^22^, markers for ER stress, were detected in *Spata7 iKO* mouse retinas (Fig. 3B, C), including an increase in CHOP-positive cells (Supplementary Fig. 1). These results show that loss of *Spata7* in adult mouse retinas causes a very similar set of phenotypes to those observed in germline *Spata7*^*−/−*^ mice.

### Loss of *Spata7* leads to microtubule destabilization in the DCC

**Figure 4:**
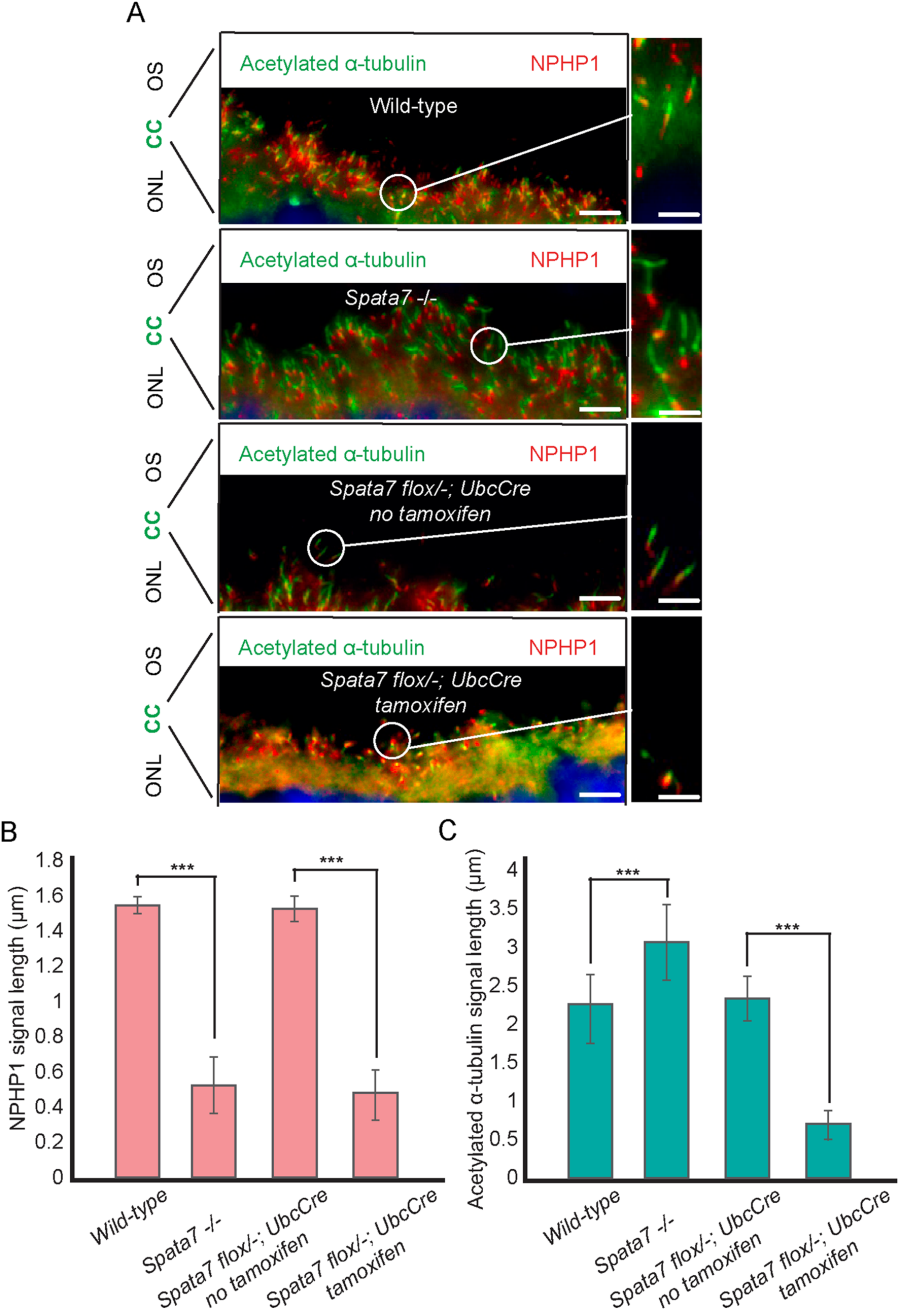
Conditional loss of *Spata7* in adult mouse retinas leads to collapse of the distal region of the CC. (**A**) Retinal cryosections of 6-month-old *Spata7*^*flox/−*^; *UbcCreERT2* mice with or without tamoxifen induction and 3-month-old wildtype and *Spata7*^*−/−*^ mice were costained for NPHP1 (red) and acetylated α-tubulin (green). A single-cilium image of NPHP1 localized to the distal CC is shown on the right. Bars for single-cilium images are 1 µm. (**B** and **C**) Histogram displaying NPHP1 (**B**) and acetylated α-tubulin (**C**) signal length from 6-month-old *Spata7*^*flox/−*^; *UbcCreERT2* mice with or without tamoxifen induction and 3-month-old *wild-type* and *Spata7*^*−/−*^ mice. Statistical analysis was performed using two-way ANOVA analysis (***p < 0.001). N = 5 mice per genotype, 10 CC per animal.

To further assess if loss of *Spata7* in the adult mouse retina leads to disruption of the CC, we performed immunostaining against acetylated α-tubulin and NPHP1. The retinal CC can be partitioned into two regions: the proximal CC (PCC), which is homologous to the transition zone (TZ) of primary cilia, and the distal CC (DCC), a photoreceptor-specific extension of the ciliary TZ^9^. Acetylated α-tubulin localizes to the tubulin-based cytoskeletal core along the entire CC^9,23^. NPHP1, a member of the NPHP–MKS complex^24^ that normally localizes along the entire CC, only localizes to the PCC in germline *Spata7* mutants^9,25^. As shown in Fig. 4A, consistent with the phenotype of germline mutants, NPHP1 signal was significantly reduced in the DCC in induced *Spata7*^*flox/−*^; *UbcCreERT2*/^+^ mice compared to controls (Fig. 4A). Both the germline knockout and adult-specific knockout mouse models showed more than a 60% reduction in NPHP1 signal length (Fig. 4B). Interestingly, significantly shortened acetylated α-tubulin signal was observed in tamoxifen-injected *Spata7*^*flox/−*^; *UbcCreERT2*/^+^ retinas (Fig. 4C). These results indicate that adult-specific knockout of *Spata7* leads to axoneme microtubule destabilization in the DCC.

## Discussion

In this study, we showed that inducible conditional knockout of *Spata7* in adult mouse retinas results in a rapid reduction in ONL thickness and decreased response to light, indicating that *Spata7* is essential for maintaining adult mouse retinal integrity and function. Combined with our previous reports, this study indicates that *Spata7* is required for both establishment and maintenance of the CC. Our findings suggest that SPATA7 is continuously required during retinal development and post-maturation. This study provides a more detailed understanding of the CC and helps clarify the overlooked roles of SPATA7 in matured retina.

In our previous report, we generated tissue-specfic *Spata7* knockout mouse models by crossing *Spata7*^*flox/flox*^ mice with photoreceptor-specific *Crx-Cre* mice or retinal pigment epithelium (RPE)-specific *Best1-Cre* mice, demonstrating sufficient and pathogenic roles of Spata7 during photoreceptor development^14^. However, the roles of Spata7 in matured photoreceptor cells remained to be elucidated. We applied the *UbcCreERT2/*^+^ mouse to generate the conditional knockout of *Spata7* in adult mouse retinas. Our results suggest that rhodopsin mislocalization and ER stress induction likely contributed to the photoceptor degeneration observed in *Spata7 iKO* mice. These phenomena are consistent with phenotypes observed in *Spata7* germline mutant mouse retinas, suggesting that the mechanism of photoceptor degeneration in *Spata7 iKO* mice involves the disruption of correct protein trafficking and induction of ER stress-mediated photoreceptor apoptosis and is similar to that of germline mutant mice. Additionally, the induction of ER stress was not limited to the photoreceptor layer. Rather, both the inner nuclear layer (INL) and the ganglion cell layer (GCL) also showed elevated ATF, P-PERK and CHOP expression. The elevated expression of ER stress markers indicates that many cell types in the retina were under stress when *Spata7* was deleted, likely a secondary effect of photoreceptor degeneration.

Previous studies indicated that SPATA7 might act as a scaffold protein that interacts with other CC proteins, such as RPGRIP1 and NPHP1, members of the RPGR and NPHP complexes^9^. RPGR and NPHP complexes act as a ciliary gate that controls access of both membrane and soluble proteins to the photoreceptor outer segment^26^. Protein–protein interactions are thought to play critical functions to maintain the integrity of microtubule filaments of the CC. The absence of SPATA7 leads to mislocalization of DCC proteins and destabilized axonemal microtubules^9^. Shortened DCC length as inferred from NPHP1 expression is observed in both *Spata7*^*−/−*^ and tamoxifen-injected *Spata7*^*flox/−*^; *UbcCreERT2*/^+^ mice. Similarly, the lengths of expression of other DCC proteins are predicted to be reduced in both germline knockout mice and adult-specific knockout mice. However/Interestingly, tamoxifen-injected *Spata7*^*flox/−*^; *UbcCreERT2*/^+^ mice and *Spata7*^*−/−*^ mice showed different microtubule patterns. In contrast to the thin and elongated axonemal microtubules observed in *Spata7*^*−/−*^ mice, axonemal microtubules in the adult *Spata7* conditional knockout mouse model were almost completely collapsed (Fig. 4A, C). The biological molecular behind this remains unknown and is worth future investigations. These data suggest that SPATA7 is continuously required for the proper structure and function of the CC, and newly synthesized SPATA7 is necessary to replenish the protein pool.

In summary, we propose that *Spata7* is also required for maintenance of the retinal CC post-maturation. Loss of *Spata7* in mature retinas results in a degeneration phenotype similar to what is observed due to loss of *Spata7* during retina development, including disruption of CC integrity and protein trafficking along microtubule tracks and activation of ER stress-mediated cell death. Additionally, given that the loss of *Spata7* in the adult retina results in more widespread ER stress in inner retina layers and more severe axonemal microtubule collapse, *Spata7* may have a larger number of requirements in mature retinas.

## Methods

### Mouse strain generation, breeding, and genotyping

This study is reported in accordance with ARRIVE guidelines (https://arriveguidelines.org). All animals were handled in accordance with the policies on the treatment of laboratory animals’ protocol (AN-4175) approved by the Institutional Animal Care and Use Committee of Baylor College of Medicine. Mice were housed on a standard diet and on a 6 am–6 pm light cycle, 20 ± 2° with 50 ± 5% relative humidity. *UbcCreERT2*/^+^ mice were purchased from the Jackson Laboratory (Stock No: 007001). *Spata7 iKO* (*Spata7*^*flox/−*^; *UbcCreERT2*/^+^) mice were generated by crossing *Spata7*^*flox/flox*^ mice with *Spata7*^*−/−*^; *UbcCreERT2*/^+^ mice. *Spata7 iKO* mice were obtained at the expected frequency (50%), and *UbcCreERT2* mice are homozygous embryonic lethal^15^. The genotyping protocols for *Spata7*^*−/−*^ and *Spata7 *^*flox/flox*^ alleles were described previously^12,14^. We used a genomic PCR assay to detect the *Spata7* allele using the following primers: P1 (5′-CACATTCATTCCCGATCTTTTTA-3) and P2 (5′-CTGACTAGGGGAGGAGTAGAAGG-3′). *Spata7* deletion was assessed by the presence of a 500 bp band after running an agarose gel.

### Tamoxifen induction

Tamoxifen (Sigma‐Aldrich, CAS#10540‐29‐1) dissolved in corn oil at a concentration of 25 mg/ml was intraperitoneally injected into adult mice (3-month-old) for 5 consecutive days. Mice were then maintained for another 3 months before retinal assays were conducted.

### Immunohistochemistry and immunostaining

Immunofluorescent staining with paraffin sections were conducted as previously described (Reference). Briefly, eyes were enucleated and fixed in modified Davidson’s fixative overnight for paraffin embedding. Eye sections (7 µm) were deparaffinized, and antigen retrieval was performed by boiling sections in 0.01 M Tris, EDTA buffer (pH 9.0) or 10 mM sodium citrate buffer (pH 6.0) for 30 min. Cooled slides were then washed in PBS, incubated for 1 h in hybridization buffer (10% normal goat serum, 0.1% Triton X-100, in PBS), and incubated with primary antibody overnight. Primary antibodies used were anti-SPATA7 (1:100, custom antibody made by Bethyl Laboratories), anti-Rhodopsin (1D4) (1:200, Santa Cruz Biotechnology sc-57432), anti-CHOP (1:200, Santa Cruz Biotechnology sc-575), anti-ATF6 (1:200, Novus Biologicals NBP1-40256), anti-P-PERK (Thr980) (1:100, Invitrogen MA5-15033), anti-NPHP1 (aa 394–687) (1:100, from Greg Pazour lab)^27^, and anti-acetylated-alpha-tubulin (1:200, Santa Cruz Biotechnology sc-23950). The next day, slides were washed in PBS, incubated with secondary antibody for 2 h, incubated with DAPI at room temperature, then mounted with anti-fade medium (Prolong; Invitrogen) and coverslipped. Fluorescent images were captured with a Zeiss Apotome.2 microscope (Zeiss Axio Imager). Relative fluorescent intensities were measured by ImageJ. All immunostaining experiments were performed independently using five biological replicates.

### ERGs

Mice were dark-adapted overnight and anesthetized with Rodent-III (22 mg/kg ketamine, 4.4 mg/kg xylazine, and 0.37 mg/kg acepromazine; intraperitoneal injection). Both pupils were dilated using tropicamide (1.0%) and phenylephrine (2.5%) and corneas were anesthetized with proparacaine (1.0%). After removing excess fluid, a drop of Goniosoft (2.5%) was placed on each cornea to keep the eye moistened and improve contact between the cornea and the ERG electrode (N1530NNC). For the scotopic recordings, luminescence at 10^−1^, 10^0^,10^1^, 10^2^ scot cd*s/m^2^ was used to record scotopic ERG a- and b-waves on a UTAS Visual Diagnostic System and EMWIN software (LKC Technologies, Gaithersburg, MD, USA). After scotopic ERG recordings, mice were light-adapted (30 cd*s/m^2^; white light) for 5 min, and then photopic ERGs were recorded with photopic luminescence of 5, 50, and 500 scot cd*s/m^2^ as previously described^28^. A minimum of 10 mice were used for each genotype for ERG analyses. Mann–Whitney tests were performed for statistical analysis (***p < 0.001).

### Quantification and data analysis

Statistical analysis of immunostaining was performed using two-way ANOVA analysis (***p < 0.001). N = 5 mice per genotype, 3 staining slides per animal. CHOP + cells were counted in the 200um region adjacent to the optic nerve. Three retinal sections were counted per animal. N = 5 mice per genotype. Error bar is SEM.

## Supplementary Information

Supplementary Figure S1.

Supplementary Figure S2.

Supplementary Legends.

## Data Availability

The datasets generated and/or analyzed during the current study are available in the [Jiaxiong] https://drive.google.com/drive/u/0/folders/12gzP93UuPV6BfDgnBt5FL3TzXwLQ9K_d.
