# Supplementary figures and images for: Spata7 is required for maintenance of the retinal connecting cilium
Source: Sci Rep. 2022 Apr 2;12:5575. doi: 10.1038/s41598-022-09530-0 (PMC8976851; doi:10.1038/s41598-022-09530-0)

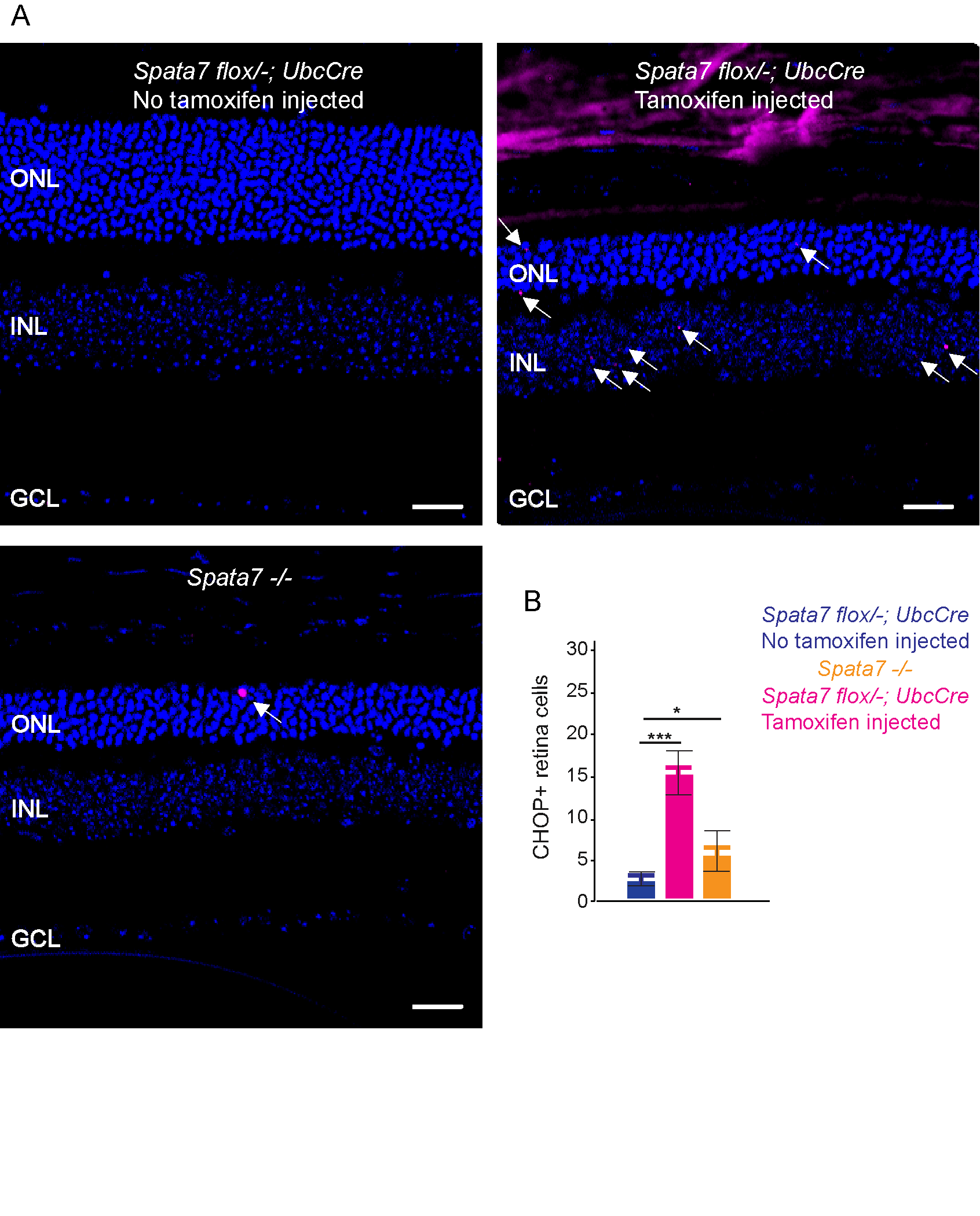

Supplement: Supplementary file 1 — Supplementary Figure S1. [file 41598_2022_9530_MOESM1_ESM.tif]

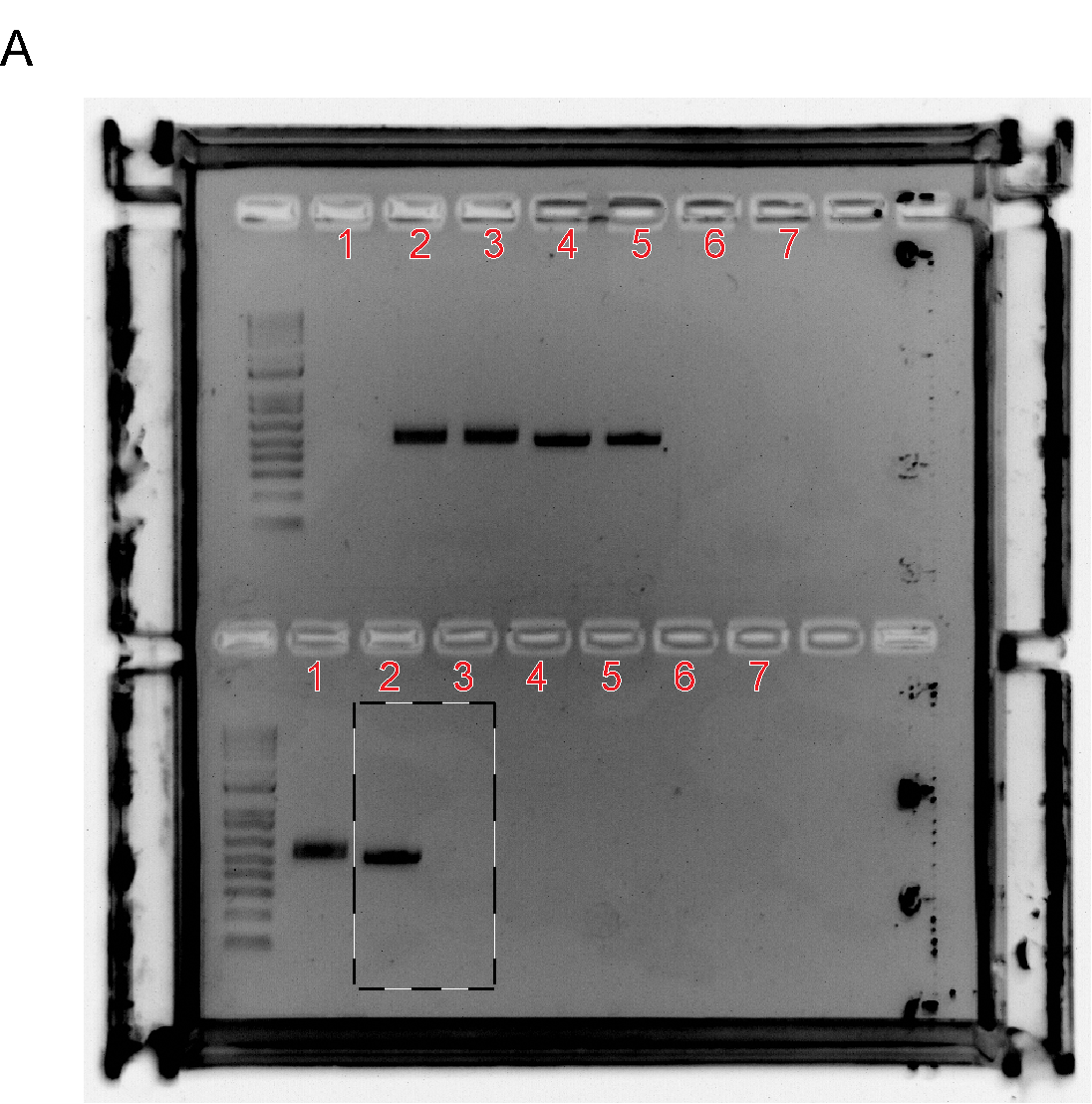

Supplement: Supplementary file 2 — Supplementary Figure S2. [file 41598_2022_9530_MOESM2_ESM.tif]
