# Supplementary material for: Spata7 is required for maintenance of the retinal connecting cilium
Source: Sci Rep. 2022 Apr 2;12:5575. doi: 10.1038/s41598-022-09530-0 (PMC8976851; doi:10.1038/s41598-022-09530-0)
Supplement: Supplementary file 3 — Supplementary Legends. [file 41598_2022_9530_MOESM3_ESM.docx]

**Supplementary Figure 1.** **Significant increase of CHOP+ cells in *Spata7 iKO* mouse retinas. (A)** A significant increase in the number of CHOP+ cells was observed in both tamoxifen-injected *Spata7^flox/-^;* *UbcCreERT2* and *Spata7^-/-^* mouse retinas compared to tamoxifen non-injected *Spata7^flox/-^;* *UbcCreERT2* retina. CHOP+ cells are marked by white arrows. Scale bar = 20 µm. **(B)** Bar plot showing quantification of CHOP+ cells. Statistical analysis was performed using the Student’s t-test and two-way ANOVA analysis (**p*<0.05 and ****p*<0.001). N = 5 mice per genotype, 3 slides (close to the optic nerve) per animal.
